# Influence of Sugars and Surface Properties on Wettability and Adhesion of Starch-Based Model Suspensions on Polytetrafluoroethylene (PTFE) and Polyethylene Terephthalate (PET) Surfaces

**DOI:** 10.3390/foods14122033

**Published:** 2025-06-09

**Authors:** Ana Caroline Frabetti, Jaqueline Oliveira de Moraes, Vanessa Jury, Lionel Boillereaux, João Borges Laurindo

**Affiliations:** 1 Department of Chemical and Food Engineering, Federal University of Santa Catarina, Engenharia Quimica et de Alimentos (EQA)/Centro Tecnologico (CTC), Florianópolis 88040-900, SC, Brazil; anacfrabetti@gmail.com (A.C.F.); jb.laurindo@ufsc.br (J.B.L.); 2 Oniris, Nantes Université, CNRS, GEPEA, UMR 6144, F-44000 Nantes, France; vanessa.jury@oniris-nantes.fr (V.J.); lionel.boillereaux@oniris-nantes.fr (L.B.); 3 Department of Food Science and Technology, University of California Davis, Davis, CA 95616, USA

**Keywords:** adhesion, polyethylene terephthalate, roughness, sugar content, polytetrafluoroethylene, wettability

## Abstract

In food drying processes such as cast-tape drying, refractance window, and drum drying, spreading food suspensions on hydrophobic surfaces is critical. This study investigated the effects of low-molar-mass sugars (glucose, sucrose, and fructose) on the rheology and surface tension of cassava starch suspensions, which served as model systems. Wettability was assessed on hydrophobic surfaces, including new polytetrafluoroethylene (PTFE) and polyethylene terephthalate (PET) films, with additional testing on sandpaper-abraded PTFE (named PTFE R+) to evaluate the influence of surface roughness. PET film exhibited lower roughness (Ra = X µm) and higher surface tension (71 mN/m) compared to PTFE (surface tension 65 mN/m). Contact angles on PET (93–124°) were significantly higher than on PTFE (89–113°), indicating greater product adhesion on PET. All suspensions showed pseudoplastic behavior, and the addition of the surfactant Tween 20 slightly reduced surface tension (by ≈1–5 mN/m) but did not significantly enhance wettability. Sucrose and fructose increased wettability on PTFE R+, but the effects of the sugar varied depending on the surface. These findings suggest that PTFE surfaces reduce product sticking during drying compared to PET. Interactions between sugars, Tween 20, and hydrophobic surfaces must be considered to optimize spreading and reduce product sticking during drying. This knowledge can guide improvements in drying processes for food products.

## 1. Introduction

During several processes like drying or cooking, fruit pulps containing low-molar-mass sugars may adhere to solid surfaces due to their high sugar concentrations. Uniform spreading on drying surfaces is critical for optimal efficiency and product quality, particularly in cast-tape and drum drying techniques. Uneven spreading can lead to inconsistencies in drying rates, product sticking, and ultimately, reduced product quality.

Several factors influence the adhesion phenomenon, including viscosity, sugar type, surface energy, and roughness, which depend on both the product’s characteristics and the solid’s surface properties. These factors can cause yield losses and difficulties in equipment cleaning. Adding carrier agents to the food suspension can reduce its adhesion to the solid’s surface, but hydrophobic surfaces are another suitable option. Using model suspensions allows us to gain a better understanding of the influence of these alternatives on food-to-surface adhesion. Recent studies on sugar-rich food drying and hydrophobic coatings in food processing have highlighted the role of surface energy in adhesion and drying efficiency. For instance, PTFE and PET have been investigated for their performance in drying systems due to their low surface energy and food-grade status [1]. Several authors reported that model food suspensions have been used to study the impact of ingredients on adhesion [2], determine state diagrams and glass transition temperature [3,4], and study drying processes and their limitations [5,6], among other applications.

Model suspensions are based on the formulation of real foods and are easily reproduced or modified, which is helpful for understanding the influence of components’ concentrations on the studied phenomena. Precise replication of real products is not required, as only key components are necessary to investigate relevant properties. These formulations help us to investigate the functionality of many food components, including starches, gums, and emulsifiers, and factors affecting the changes occurring during industrial processing (lipid oxidation, Maillard reaction, etc.). Therefore, model suspensions provide a means of determining how the components of a product and processing can change the characteristics of the final product. They help assess the sensitivity of a given food to different ingredients and processing steps [7].

A typical fruit model suspension can be formulated by adding sugars to a basic formulation. In these foods, the sugar content ranges from negligible (e.g., avocado) to more than 20% (e.g., ripe banana) by mass of wet matter. Sucrose, glucose, and fructose are the main sugars found in most fruits. Generally, fruits and vegetables contain more reducing sugars than sucrose [8].

One of the main challenges of drying food by heat conduction is predicting the interaction between the liquid suspension (i.e., fruit pulp) and a heated solid surface, considering that this suspension must wet the material’s surface to spread it homogeneously and withdraw the dried product. Therefore, it is essential to identify the adhesion phenomena involved during the spreading and drying of food suspensions on heated surfaces. Surface tension and wettability measurements, including the effect of the surface’s roughness, are needed to understand surface–food interactions that occur during drying in industrial systems. In such a manner, it is possible to predict the interaction between a liquid and an equipment surface more accurately. Multiple factors influence the interactions, depending on the suspension characteristics (composition, viscosity, surface tension) and the solid’s roughness and wettability [9,10].

The liquid–solid surface interaction, assessed based on the wettability, is crucial for drying processes. Wettability, which is related to the surface and liquid energy, is assessed via the contact angle (θ) formed between the liquid and solid. A contact angle below 90° indicates a hydrophilic surface, while a contact angle above 90° indicates hydrophobicity. These properties influence drying efficiency and product removal: hydrophilic surfaces promote liquid spreading, whereas hydrophobic ones may facilitate detachment after drying. For example, PTFE surfaces are often used for drying because their hydrophobic properties prevent the dried product from sticking, facilitating easy removal [11].

The factors favoring wettability (e.g., a small contact angle) are the significant adhesive force between liquid and solid and the relatively low cohesive force (surface tension) within the liquid. As a result, the adhesive forces between a liquid and a solid surface can be changed by dropping the liquid cohesive forces with surfactant compounds. The other option for achieving higher wettability is selecting a solid with a high surface energy [12,13].

Metals are widely used in food processing for their durability and ease of cleaning, although they have high surface tension, which favors adhesion, especially for high-sugar-content products. Otherwise, polymers such as PET or PTFE-coated fiberglass have low surface tension. Therefore, water solutions and suspensions generally show greater wettability in metals than in polymers. PTFE-coated tapes are considered food-grade, and their use has increased in the food industry. This is because these surfaces have low interactions with foods, even at high temperatures, up to 260 °C. However, its durability depends on its use condition, which can produce surface roughness by the natural aging and scraping associated with product removal or cleaning procedures [14].

PTFE and PET were chosen for their food-grade status, low surface energy, and widespread use in drying equipment. Recent studies have demonstrated the effectiveness of PTFE and PET in enhancing drying processes due to their hydrophobic properties [15,16]. Therefore, the objective of this study was to determine how starch-based model suspensions interact with brand new hydrophobic surfaces, and with artificially created roughness to simulate their long-time use. For this purpose, a PTFE-coated tape (brand new, Teflon^®^, and with an abraded surface, named PTFE R+), and a commercial PET film were employed as surface materials. In addition, the rheology of the fruit model suspensions and their contact angles with these three different surfaces were investigated to understand their influence on the material adhesion and detachment after drying.

## 2. Materials and Methods

A 0.23 mm thick fiberglass fabric with 56% of polytetrafluoroethylene (Teflon^®^, Indaco, Sheet Armalon^®^ Standard, São Paulo, Brazil) and a 0.25 mm thick polyethylene terephthalate film (DuPont^®^, Mylar type D, Wilmington, DE, USA) were used as hydrophobic surfaces for the spreading of the model suspensions.

Starch is a predominant food reserve substance in plants and does not interact strongly with the added sugars that were used to complete the model suspensions. Therefore, cassava starch (Yoki Alimentos S.A., São Bernardo do Campo, Brazil) was the main compound used to form the model suspensions. D (−) Fructose PA 99.99% pure (molar mass 180.16 g mol^−1^), D (+) anhydrous glucose PA 99.50% pure (molar mass 180.16 g mol^−1^), and sucrose of analytic grade (molar mass 342.30 g mol^−1^) of Sigma-Aldrich, St. Louis, MO, USA were used as low-molar-mass sugars.

Statistical analyses of the results were performed using Statistica software (version 13.5, TIBCO Software Inc., 2018, Palo Alto, CA, USA). Statistical analyses were performed using one-way ANOVA followed by Student’s *t*-test and Tukey’s post hoc test, depending on the comparison.

### 2.1. Roughness of PTFE and PET Surfaces

The influence of different amounts of surface roughness in the solid–liquid interaction was studied by scraping the PTFE-coated fabric with a 180-grit sandpaper (3M, São Paulo, Brazil). The sandpaper was fixed in an automatic spreader and slid ten times over the surface of the PTFE, creating grooves. The surface with modified roughness was called PTFE R+. Afterwards, the materials were cut into specimens. A white light optical interferometer (NV 7300, Zygo NewView 7300, Zygo Corporation, Middlefield, CT, USA) was used for measurements of non-contact surface topography on the brand new material (unused PTFE without abrasion) and on the surface that was subjected to abrasion, PTFE R+. Mean surface roughness (Sa) results, expressed in μm, were obtained after at least three measurements were collected in different regions of each sample area. Furthermore, the mean roughness of PET film, measured by an optical profilometer, was obtained from the supplier’s database [17].

### 2.2. Surface Tension of PTFE and PET

The contact angles of three pure liquids (water, glycerol, and diiodomethane) on the PTFE, PTFE R+, and PET film were measured with a Drop Shape Analyzer (DSA25, KRÜSS Scientific, Germany) at 20 °C, according to Michalski and co-authors [18]. Measurements were performed at 20 °C, and an average of at least five repetitions was calculated. Ethanol was used to clean the solid materials for proper analysis. The thermodynamic work of adhesion, $W_{A}$, can be calculated using Young–Dupré’s equation (Equation (1)) from the measured properties, $\gamma_{L}$ and $\theta_{0}$. Young–Dupré’s equation was applied to calculate the solid surface tension ($\gamma_{S}$).

(1) $$W_{A}=\gamma_{L}(1\;+\cos\theta_{0})$$

The Lifshitz–van der Waals ($\gamma_{S}^{LW}$) and acid–base ($\gamma_{S}^{+}$ and $\gamma_{S}^{-}$) components can be calculated via the Van Oss model, as shown in Equation (2):

(2) $$W_{A}=2{\left(\gamma_{L}^{LW}\gamma_{S}^{LW}\right)}^{1/2}+2{\left(\gamma_{L}^{+}\gamma_{S}^{-}\right)}^{1/2}+2{\left(\gamma_{L}^{-}\gamma_{S}^{+}\right)}^{1/2}$$

where $W_{A}$ is the work of adhesion (in J m^−2^ or N m^−1^), $\gamma_{L}$ is the surface tension of the liquid (in mN m^−1^), θ_0_ is the contact angle of the liquid with the substrate, and $\gamma_{S}^{LW}$ is the Lifshitz–van der Waals component, while $\gamma_{S}^{+}$ and $\gamma_{S}^{-}$ are the acid–base components.

### 2.3. Preparation of the Fruit Model Suspensions

**Figure 1:**
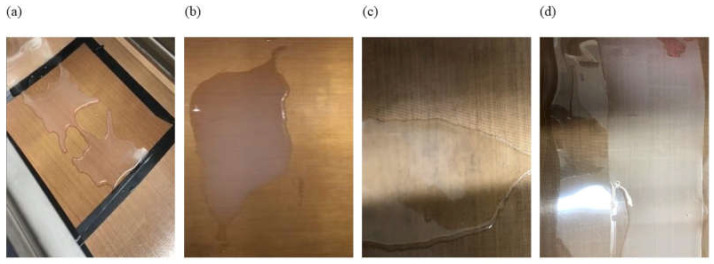
Spreading of model suspensions composed of (**a**) water + 1.98 g of glucose, fructose, and sucrose on PTFE; (**b**) 4% cassava starch on PTFE; (**c**) water + 1.98 g of glucose, fructose, and sucrose + beet fiber on PTFE; (**d**) 4% cassava starch + 1.98 g of glucose, fructose and sucrose on PET. Note: This figure is used as a visual support to justify the use of Tween 20 to ensure spreading.

The cassava starch suspension was prepared from 4 g per 100 g of water. Starch gelatinization was performed in an ultra-thermostatic water bath (MAXIM-LAB, Brazil) at 71 °C, for 5 min, under mechanical stirring (IKA RW20 digital, Germany) at 210 rpm. Previous experiments, presented in Figure 1, have shown that the spreading of the model suspensions was impossible on PTFE and PET surfaces.

This problem was solved by adding 0.1% (volume/mass of suspension) of surfactant Tween 20 to the model suspensions. The amount of Tween was defined in preliminary testing, which allowed us to determine the minimum volume of surfactant required to spread the fruit model suspensions on hydrophobic surfaces without promoting micelle formation, which could alter surface tension significantly [19].

The sugars were added to 100 g of starch suspension at 65 °C under constant stirring, using a magnetic stirrer, until the solutes were completely dissolved. The sugar concentrations used (3–6 g/100 g suspension) were selected to mimic real fruit sugar levels, which generally range from 5 to 20% (wet basis) [20]. The formulations of low-molar-mass sugars are presented in Table 1.

### 2.4. Rheology of the Fruit Model Suspensions

The flow properties of the model suspensions were determined using a Haake Mars rotary rheometer (Modular Advanced Rheometer System, Thermo Scientifc^®^, Germany) with parallel plate geometry. A 60 mm diameter plate (PP60) and 3 mL of solution were used.

Viscosity curves were determined with a rotation ramp of 0.001 s^−1^ to 600 s^−1^ for 180 s, completed with the reverse path, also performed in 180 s. During the experiment, the temperature was controlled by a water bath at 21 ± 2 °C.

The Ostwald (Equation (3)) and Herschel–Bulkley (Equation (4)) models were applied to the shear stress (σ) versus shear rate ($\dot{\gamma }$) curves for all the samples:

(3) $$\sigma =K{\dot{\gamma }}^{n}$$

(4) $$\sigma =\sigma_{0}+K{\dot{\gamma }}^{n}$$

where K is the consistency index (Pa·s^n^), n is the flow behavior index, and $\sigma_{0}$ is the initial shear stress.

### 2.5. Surface Tension of the Fruit Model Suspensions

The surface tension of the model suspensions without surfactants and with the addition of 0.1% (volume/mass of suspension) of Tween 20 were measured using a tensiometer KRÜSS K12 (KRÜSS GmbH, Hamburg, Germany). Ten measurements were taken for each suspension, and the measurements were repeated at least twice. The surface tension (T) results were expressed in mN m^−1^.

### 2.6. Contact Angle of the Fruit Model Suspensions on PTFE and PET

The contact angle measurements of the model suspensions were performed on a Ramé-Hart goniometer (model 250, Succasunna, NJ, USA) using the sessile drop method. A 5 µL drop of each liquid was placed on the surfaces (PTFE, PTFE R+, and PET) with an automated micropipette and the contact angles were assessed with a camera.

## 3. Results

### 3.1. Surface Roughness and Surface Tension of PTFE and PET

The mean roughness values (Sa) obtained for brand new PTFE and PTFE R+ (with abrasions) are shown in Table 2, along with the PET mean roughness value reported by the material’s supplier [17]. The same table also presents the calculated values of the solid surface tension $\gamma_{S}$ and its components $\gamma_{S}^{LW}$, $\gamma_{S}^{+}$ and $\gamma_{S}^{-}$ obtained from contact angle measurements using the Van Oss model (Equation (2)) and three probe liquids (water, glycerol, and diiodomethane).

PET exhibited a smoother surface compared to both PTFE samples. The unused PTFE showed a smaller value of roughness compared to the PTFE R+, although there was no statistically significant difference between them. This is because the grooves were created at specific points on the materials, and the “Sa” parameter expresses the average of absolute values in the analyzed area. Thus, the roughness is the result of the arithmetic mean of this property in the measured region of the three-dimensional display diagram. Therefore, the influence of a single scratch on the measurement value is relatively small [21].

The roughness of a certain surface plays a crucial role in drying processes. A rougher surface provides more nucleation sites for droplet formation, which can enhance heat and mass transfer. Additionally, a rougher surface can increase the contact area between the liquid and the solid, improving wetting and promoting faster drying. However, excessive roughness can also hinder drying by trapping liquid in crevices, leading to uneven drying and potential product defects [22,23].

Furthermore, Table 2 displays the contact angles of diiodomethane, glycerol, and water on all the tested surfaces and the surface tension components obtained using the Van Oss model (Equation (1)). PTFE exhibited water contact angles greater than 100°, indicating its hydrophobic nature. [24]. The results showed that the PET surface is less hydrophobic, with smaller contact angles with water. Also, the abrasion with sandpaper (PTFE R+) caused a decrease in the contact angle. Karim and others [25] evaluated the spread of polyethylene glycol (PEG) solutions on PTFE plates with varying levels of roughness (305, 86, and 38 µm) and reported equilibrium contact angles between 100° and 120°. The authors also noticed that the roughness of PTFE surfaces had a significant effect on reducing contact angles.

Because of its inertness, PTFE is expected to interact weakly and non-preferentially with liquids, which explains the high contact angles with all tested liquids. Diiodomethane, a hydrophobic liquid with a chemical nature closer to PTFE, interacted a bit more strongly with the support [26]. A surface with a hydrophobic character is also characterized by a lower $\gamma_{S}^{LW}$, with little (or zero) Lewis’s acid or Lewis’s base character. On the other hand, a surface may be hydrophilic because of the presence of Lewis’s base or acid groups, or both. It is evident that changes in the surface structure that increase, for example, the concentration of ether groups, will increase Lewis’s base character, and hence its hydrophilicity, with little influence on the character of Lewis’s acid [27].

As expected, the PTFE surface with its original roughness showed lower surface tension, similar to that reported in the literature [24,28], but this property increased with the increase in the PTFE roughness. For PET, the smaller contact angle with water demonstrates the more hydrophilic character of this material.

The interplay between surface roughness and surface tension is crucial for optimizing food drying processes that rely on spreading food suspensions. Thus, the findings on how surface tension and roughness (including modified PTFE R+) affect the wettability of a food solution can inform the selection of appropriate drying surfaces for different food products. Despite the initial high wettability, the strong attraction between PET and water (hydrophilic character) can lead to increased sticking of food components, especially those with high water content. This can make it difficult to remove the dried food material from the surface. For PTFE, its low initial wettability and non-stick properties generally lead to reduced sticking of food solutions, allowing the product to be released more easily after drying. The increase in roughness of this hydrophobic material created more contact points for the food model solution, potentially leading to increased sticking compared to the smooth PTFE surface.

### 3.2. Rheological Behavior of the Fruit Model Suspensions

**Figure 2:**
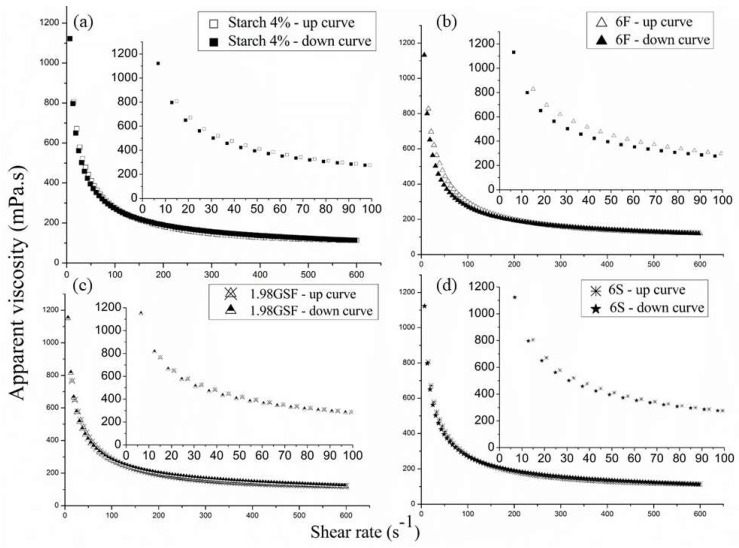
Viscosity vs. shear rate curves for the fruit model suspensions at 21 ± 2 °C. The inset in each graph highlights the initial decay of viscosity at shear rates between 0 and 100 s^⁻1^: (**a**) suspension of cassava starch 4%; (**b**) suspension 6F (6 g fructose/100 g suspension); (**c**) suspension 1.98GSF (1.98 g glucose + 1.98 g sucrose + 1.98 g fructose/100 g suspension); (**d**) suspension 6S (6 g sucrose/100 g suspension).

Only the rheological behavior of selected suspensions is shown in Figure 2, as the behaviors of other model groups were similar. The rheological behavior of the other suspensions can be found in the Supplementary Material. 

All model suspensions showed pseudoplastic behavior, as their apparent viscosities decreased with the increase in the shear rate. Other authors reported similar results in studies with starch suspensions [29,30,31,32]. This shear-thinning nature facilitates better spreading on surfaces at low shear rates, which is advantageous for forming uniform films. The curves were very similar, exhibiting an identical decay, which cannot be attributed to the different compositions of the model suspensions.

Table 3 shows Ostwald and Herschel–Bulkley’s parameters determined from these models to fit the shear stress vs. shear rate curves.

The flow index (n) values ranged between 0 and 1, while K values were always greater than zero (K > 0), which confirms the suspensions’ pseudoplastic behavior.

Both models fit well with the sample data, with high coefficients of determination (R^2^). The Herschel–Bulkley model, in particular, had an R^2^ greater than 0.99 for all the sugar compositions. The adjustment of the Hershel–Bulkley model to the experimental data showed that the suspensions presented an initial shear stress $\sigma_{0}$ for the beginning of the flow, which indicates the relationship with the samples’ sugar composition. The initial shear stress $\sigma_{0}$ was lower for suspension 3S3F and higher for suspension 3G3F, although the values are similar for all samples. Adding sugars increased the Herschel–Bulkley model’s parameters, increasing the consistency (K) and flow (n) indexes. However, for n, there were no significant differences compared to the sample containing only starch. Other authors reported an increase in the initial shear stress $\sigma_{0}$ and in the K value with the addition of sugars to starch suspensions [33]. Abu-Jdayil and co-authors [34] investigated the effect of sugars on the rheological properties of starch pastes and reported a resulting pseudoplastic behavior for the suspensions. This pseudoplastic behavior can influence the drying process, as it affects the spreadability of the suspension on the surface. For instance, a more pseudoplastic suspension may spread more easily, leading to a thinner film and potentially faster drying.

### 3.3. Surface Tensions of the Fruit Model Suspensions

Table 4 displays the surface tensions (T) of the model suspensions without adding surfactants and with 0.1% of Tween 20.

The surface tension of the suspension containing 4% starch, without sugars, resulted in a surface tension close to that of water (72 mN m^−1^). The results showed a significant statistical difference, according to Tukey’s test, between the 4% starch suspension and some of the suspensions containing sugars.

For most suspensions, except 3G3F, the addition of surfactant did not result in a significant difference in surface tension values when compared to the same sugar composition without surfactant, according to Student’s *t*-test. These results demonstrated that adding surfactant did not strongly influence the surface tension of the starch-based suspensions. This is likely due to the rapid micellization of Tween 20 in aqueous media [19], which may limit its surface activity at such low concentrations. However, a slight trend of lower values could be observed when Tween 20 was present (disregarding statistical analyses). It is usual to add emulsifiers to suspensions containing sugars and low-molar-mass polysaccharides before drying to improve their stability and promote homogeneous particle distribution. Surfactants (such as Tween) added to food suspensions usually decrease the surface tension of the aqueous phase (reducing the contact angle) and enhance the interaction between the suspension and the surface [19]. In our study, Tween 20 was added to the suspensions to allow the surface to be wet while spreading the suspensions. The amount of surfactant added was small, so the results were not considerably impacted. Also, Tween has fast micellization in water, which may be the reason for its higher surface tension (close to the value of water) when present in small amounts in solutions [19]. The presence of surfactants in suspensions can increase their wettability in certain materials, mainly hydrophobic surfaces and those that have undergone modifications [35,36].

Oroian and co-authors [37] evaluated the surface tension of sucrose, glucose, and fructose solutions, using different temperatures and concentrations in water. The results showed that, at ambient temperature (25 °C), changes in sugar concentration had little influence on the surface tension.

### 3.4. Contact Angle of the Fruit Model Suspensions of PTFE and PET

Table 5 summarizes the contact angles measured for all fruit model suspensions, detailing the influence of sugar composition on wettability across three types of surfaces: smooth PTFE (new), roughened PTFE (PTFE R+), and PET (new).

In general, all the suspensions exhibited greater contact angles on PTFE than on PET. The literature refers to the greater wettability of a suspension on a particular surface as the determinant for greater adhesion of the dry product to that surface [38,39]. The contact angle between suspensions and PET ranged from 72.9 to 92.6°, pointing out the influence of the formulation when a high interaction liquid surface is observed. For unused PTFE, the contact angles ranged from 99.1 to 102.9°. In an evaluation of the wettability of the model suspensions on PTFE and PTFE R+, it was noted that the PTFE surface, with its original roughness, presented greater contact angles for most suspensions, in comparison to the PTFE R+. These results agree with discussions available in the literature that state that the wettability of a solution is altered by changing the roughness of the contact material [13,40,41,42]. The PTFE surface is composed of -CF2- bonds fragments forming an inert structure with low surface tension. Therefore, the contact angle of PTFE surfaces is above 100°. When the PTFE surface is roughened, the -CF2- bonds may be broken, and carbon–hydrogen and carbon–oxygen bonds are formed. So, the hydrophobic property of the non-roughened surface (-CF-) is attenuated (-CH- or -C-O-), decreasing the contact angle [43].

As the PTFE sample is a fiberglass fabric with PTFE-coated material, this covering layer may have been removed by the sandpaper abrasion, exposing the fiberglass and increasing the surface wettability.

The results shown in Table 5 confirm the hypothesis that the wettability is affected by the support’s roughness and by the composition of the spread suspension. Michalski and others [18] studied the adhesive behavior of food emulsions on PTFE and other surface materials. They stated that surface roughness and fluid properties are the main factors influencing adhesion. The composition of the suspensions, especially the type of sugars, significantly influenced their rheological behavior and wettability. For instance, sucrose—with its higher molar mass (342 g/mol) compared to fructose (180 g/mol)—may have increased the suspension viscosity, which can reduce wettability on hydrophobic surfaces such as PTFE. Indeed, suspensions containing sucrose exhibited higher contact angles on PTFE, indicating lower affinity between the suspension and the surface.

The use of PTFE or PET was more significant in terms of the contact angle results than the suspensions’ formulation. For PTFE, sucrose had a more substantial influence on increasing the contact angle. On the PET surface, the individual sugars and the mixture of sucrose and glucose promoted a lower contact angle, leading to superior wettability. The more important aspect to consider seems to be the interaction between the suspensions and a given surface, despite the influence of sugar concentration on contact angles and wettability. Concerning improved roughness, the presence of this characteristic results in greater wettability and, consequently, stronger product adhesion. However, on PET surfaces with lower roughness, the suspensions always showed greater wettability, which proves the effect of the material surface in this phenomenon.

These findings have implications for drying applications. Improved wettability often correlates with stronger adhesion of dried residues, especially on hydrophilic surfaces like PET. In contrast, the high contact angles observed on PTFE suggest lower spreading and potentially reduced adhesion of dried material—properties which are favorable for non-stick drying surfaces. On roughened PTFE (PTFE R+), increased surface area promoted more interaction with the suspensions, resulting in reduced contact angles and likely stronger adhesion.

## 4. Conclusions

Studies to reduce food adhesion during processing are crucial for the design of efficient drying equipment. However, modifications to dryer surfaces remain rare, as materials are primarily chosen based on their thermal properties, hardness, and compatibility with cleaning protocols. Surface tension and material roughness are rarely considered in the layout of drying equipment [10]. On the other hand, food composition can be modified quickly by adding hydrocolloids or decreasing the low molar sugar concentration. Ideally, the use of hydrophobic surfaces (materials with low surface energy), coupled with the control of the liquid’s wettability and viscosity at the processing temperature, is the way to achieve the best processing condition.

In this study, surface characterization revealed that untreated PTFE exhibited hydrophobic properties and low surface energy, which contributed to reduced liquid adhesion. In contrast, abraded PTFE (PTFE R+) displayed higher surface energy and increased wettability, likely leading to greater adhesion. PET, while smoother than PTFE, showed higher wettability, potentially promoting stronger interactions with liquid suspensions.

Model suspensions were useful for evaluating the influence of sugar composition on wettability and adhesion. All formulations exhibited pseudoplastic behavior and were able to spread across the PTFE surfaces. The presence of surfactant facilitated spreading without significantly altering surface tension values. Notably, the sucrose-only suspension (6S) demonstrated lower wettability on new PTFE, suggesting a lower risk of initial adhesion.

It is important to note that adhesion also depends on temperature and moisture content, which evolve during drying. As a result, adhesion at the end of the drying process may differ significantly from the initial wettability of the liquid suspension. This makes it difficult to predict dry film adhesion from wetting measurements alone.

The absence of drying trials within the present study represents a limitation in directly correlating wettability with adhesion under industrial conditions. Nevertheless, this limitation is partially addressed in a future study, where the same model suspensions were dried using cast-tape drying (CTD) technology, and the detachment forces of the resulting dry films from PTFE and PET surfaces were quantitatively evaluated.

These findings suggest that unmodified PTFE surfaces may reduce adhesion during drying. Our research group also studies actual fruit pulps, evaluating the effect of temperature on wettability and drying behavior, and validating laboratory observations with pilot-scale drying experiments.

## Figures and Tables

**Table 1:** Sugar composition of the fruit model suspensions.

Model Suspensions	Glucose (g/100 g Suspension)	Sucrose (g/100 g Suspension)	Fructose (g/100 g Suspension)
Starch 4%	0	0	0
3G3F	3	0	3
6F	0	0	6
3G3S	3	3	0
6G	6	0	0
1.98GSF	1.98	1.98	1.98
3S3F	0	3	3
6S	0	6	0

**Table 2:** Mean surface roughness, equilibrium contact angles and calculated surface tensions of PTFE and PET materials at 20 °C.

Supports	Mean Surface Roughness (µm)	Contact Angle (°)			Surface Tension Components (mJ m^−2^)			
		DIM	Glycerol	Water	$\gamma_{S}$	$\gamma_{S}^{LW}$	$\gamma_{S}^{+}$	$\gamma_{S}^{-}$
PTFE—new	0.174 ± 0.05 ^a^	92.7 ± 4.3 ^a^	105.4 ± 2.5 ^a^	124.0 ± 1.2 ^a^	18.67	9.78	3.88	5.09
PTFE—(R+)	0.213 ± 0.02 ^a^	89.5 ± 4.4 ^a^	105.7 ± 1.8 ^a^	113.8 ± 2.5 ^a^	59.82	8.33	18.69	35.46
PET—new	0.038 ^1^	43.5 ± 2.0 ^b^	75.4 ± 2.8 ^b^	93.5 ± 1.3 ^b^	132.05	26.60	40.07	69.38

^1^ [17]. Means with different superscript lowercase letters in the column indicate that the values present a significant difference at a 95% confidence level by Tukey’s test.

**Table 3:** Adjustment of shear stress vs. shear rate curves of the fruit model suspensions to the Ostwald and Herschel–Bulkley models.

	Ostwald Model (Power Law)			Herschel–Bulkley Model			
Model Suspensions	K [Pa·s^n^]	n	R^2^	$\sigma_{0}$ [Pa]	K	n	R^2^
Starch 4%	2.575 ± 0.361 ^a^	0.515 ± 0.035 ^a^	0.985	13.78 ± 1.181 ^ab^	0.370 ± 0.113 ^cd^	0.795 ± 0.064 ^ab^	0.993
3G3F	2.520 ± 0.424 ^a^	0.530 ± 0.028 ^a^	0.986	14.61 ± 0.460 ^a^	0.350 ± 0.127 ^d^	0.815 ± 0.064 ^a^	0.993
6F	2.455 ± 0.460 ^a^	0.535 ± 0.021 ^a^	0.991	11.95 ± 0.969 ^abc^	0.520 ± 0.113 ^bcd^	0.750 ± 0.028 ^ab^	0.996
3G3S	2.200 ± 0.198 ^a^	0.540 ± 0.014 ^a^	0.994	9.125 ± 0.233 ^cd^	0.680 ± 0.057 ^abc^	0.710 ± 0.014 ^ab^	0.997
6G	2.640 ± 0.269 ^a^	0.525 ± 0.007 ^a^	0.994	11.21 ± 1.280 ^bc^	0.695 ± 0.007 ^ab^	0.710 ± 0.000 ^ab^	0.998
1.98GSF	2.095 ± 0.163 ^a^	0.560 ± 0.014 ^a^	0.990	12.69 ± 0.629 ^ab^	0.365 ± 0.007 ^d^	0.810 ± 0.000 ^a^	0.996
3S3F	2.225 ± 0.049 ^a^	0.530 ± 0.000 ^a^	0.995	7.355 ± 0.233 ^d^	0.865 ± 0.006 ^a^	0.660 ± 0.014 ^b^	0.998
6S	2.470 ± 0.014 ^a^	0.525 ± 0.007 ^a^	0.989	12.72 ± 0.693 ^ab^	0.445 ± 0.035 ^bcd^	0.770 ± 0.014 ^ab^	0.996

Means with different superscript lowercase letters in the column indicate that the values present a significant difference at a 95% confidence level according to Tukey’s test.

**Table 4:** Surface tension of the fruit model suspensions without Tween and with 0.1% Tween 20.

Model Suspensions	T (mN m^−1^)	
	without Tween 20 ^1,2^	with 0.1% Tween 20 ^1,2^
Starch 4%	71.47 ± 1.908 ^aA^	70.34 ± 2.738 ^aA^
3G3F	69.67 ± 1.493 ^aA^	64.72 ± 1.805 ^dB^
6F	70.94 ± 1.269 ^aA^	69.11 ± 2.087 ^abA^
3G3S	70.55 ± 1.443 ^aA^	67.78 ± 1.873 ^abcA^
6G	66.19 ± 1.435 ^bA^	65.92 ± 1.754 ^cdA^
1.98GSF	66.68 ± 1.090 ^bA^	65.29 ± 1.646 ^cdA^
3S3F	65.64 ± 0.494 ^bA^	67.76 ± 1.850 ^abcA^
6S	65.85 ± 0.565 ^bA^	67.20 ± 1.853 ^bcdA^

^1^ Means with different superscript capital letters on the line indicate that the values present a significant difference at a 95% confidence level by Student’s t-test. ^2^ Means with different superscript lowercase letters in the column indicate that the values present a significant difference at a 95% confidence level by Tukey’s test.

**Table 5:** Contact angles between starch-based suspensions on PTFE and PET supports.

Suspensions	Starch 4% ^1,2^	3G3F ^1,2^	6F ^1,2^	3G3S ^1,2^	6G ^1,2^	1.98 GSF ^1,2^	3S3F ^1,2^	6S ^1,2^
Surface	Contact Angle (°)							
PTFE—new	99.4 ± 4.1 ^a,CD^	99.1 ± 3.7 ^a,D^	99.1 ± 2.6 ^a,CD^	100.4 ± 1.9 ^a,BC^	100.0 ± 2.4 ^a,CD^	102.0 ± 2.2 ^a,AB^	99.5 ± 1.3 ^a,CD^	102.9 ± 2.2 ^a,A^
PTFE R+	99.5 ± 2.7 ^a,AB^	96.3 ± 3.7 ^b,C^	98.7 ± 2.8 ^a,B^	101.1 ± 2.9 ^a,A^	98.7 ± 1.5 ^b,B^	93.3 ± 8.7 ^b,D^	94.6 ± 3.7 ^b,CD^	94.3 ± 1.8 ^b,CD^
PET—new	92.6 ± 3.9 ^b,A^	85.1 ± 4.7 ^c,CD^	86.2 ± 3.2 ^b,BC^	72.9 ± 6.3 ^b,G^	80.0 ± 3.8 ^c,EF^	79.2 ± 3.3 ^c,F^	82.5 ± 6.5 ^c,DE^	88.7 ± 4.2 ^c,B^

^1^ Means with different superscript capital letters on the line indicate that the values present a significant difference at a 95% confidence level, according to Tukey’s test. ^2^ Means with different superscript lowercase letters in the column indicate that the values present a significant difference at a 95% confidence level, according to Tukey’s test.

## Data Availability

The original contributions presented in this study are included in the article/Supplementary Materials. Further inquiries can be directed to the corresponding author.

## Supplementary Materials

The following supporting information can be downloaded at https://www.mdpi.com/article/10.3390/foods14122033/s1, Figure S1. Viscosity vs. shear rate curves for the fruit model suspensions at 21 ± 2 °C. The inset in each graph highlights the initial decay of viscosity at shear rates between 0–100 s⁻¹: (a) Suspension 3G3F (3 g fructose + 3 g glucose/100 g suspension) (b) Suspension 3G3S (3 g fructose + 3 g sucrose/100 g suspension); (c) Suspension 6G (6 g glucose/100 g suspension); (d) 3S3F (3 g sucrose + 3 g fructose /100 g suspension).
