# Supplementary material for: Influence of Sugars and Surface Properties on Wettability and Adhesion of Starch-Based Model Suspensions on Polytetrafluoroethylene (PTFE) and Polyethylene Terephthalate (PET) Surfaces
Source: Foods. 2025 Jun 9;14(12):2033. doi: 10.3390/foods14122033 (PMC12192528; doi:10.3390/foods14122033)

Supplementary Figure. Viscosity vs. shear rate curves for the fruit model suspensions at 21 ± 2 °C. The inset in each graph highlights the initial decay of viscosity at shear rates between 0–100 s^{-1}: (a) Suspension 3G3F (3 g fructose + 3 g glucose/100 g suspension) (b) Suspension 3G3S (3 g fructose + 3 g sucrose/100 g suspension); (c) Suspension 6G (6 g glucose/100 g suspension) ; (d) 3S3F (3 g sucrose + 3 g fructose /100 g suspension)

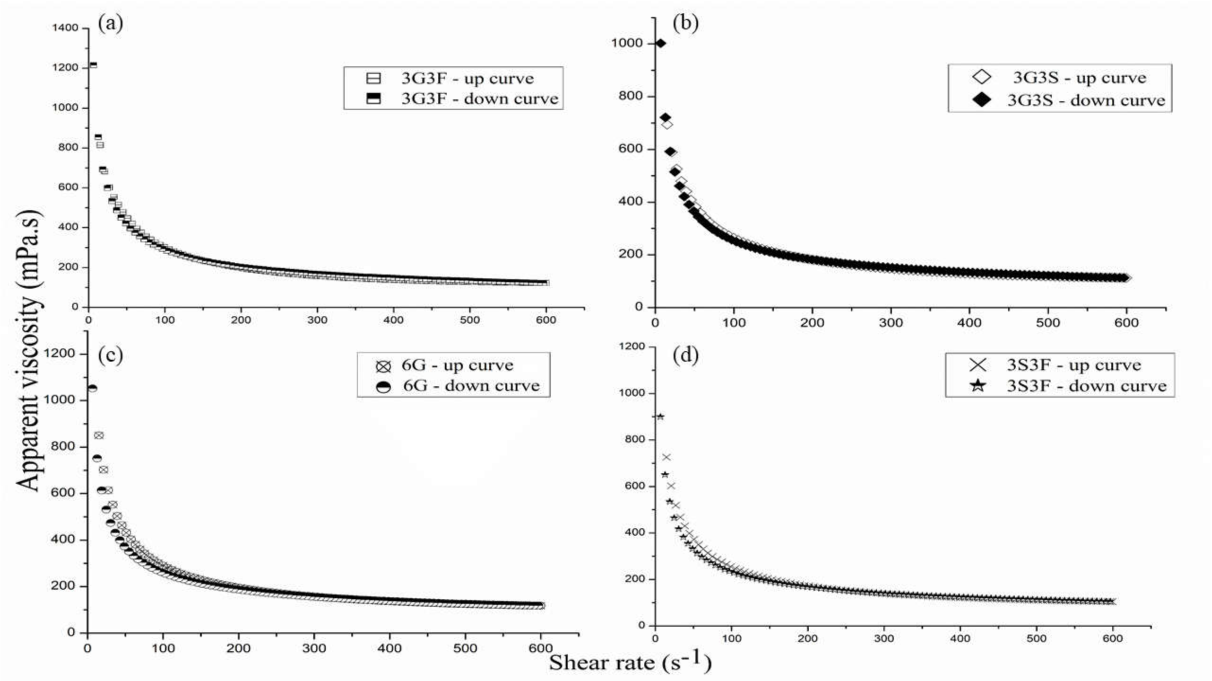

Supplement: Supplementary file 1 [file foods-14-02033-s001.zip › Figures - supplementary material.pdf]
